# Harnessing the gut microbiome for precision therapeutics in heart failure

**DOI:** 10.3389/fphar.2026.1781470

**Published:** 2026-04-15

**Authors:** Jiayue Li, Shaoheng Zhang, Yuliang Zhang, Xinlei Wang, Yantong Zhuge, Qidong Wu, Yilin Zhao, Qi Gao, Ran Chen, Yiru Wang, Qipeng Jin, Yiyi Zhang

**Affiliations:** Department of Critical Care Medicine, Longhua Hospital, Shanghai University of Traditional Chinese Medicine, Shanghai, China

**Keywords:** gastrointestinal microbiome, heart failure, metabolome, pharmacomicrobiomics, precision medicine

## Abstract

Heart failure (HF) management remains challenging because patients often show large differences in how well treatments work and in how often adverse drug reactions occur. Traditional pharmacogenomics cannot fully explain these differences. Emerging evidence from pharmacomicrobiomics shows that the gut microbiome represents a previously underappreciated factor influencing drug responses. This review summarizes the two-way interactions between the gut microbiota and key HF drugs, including digoxin, angiotensin receptor-neprilysin inhibitors (ARNIs), ACE inhibitors (ACEIs), angiotensin receptor blockers (ARBs), β-blockers, sodium-glucose cotransporter 2 (SGLT2) inhibitors, mineralocorticoid receptor antagonists (MRAs), and diuretics. On the one hand, gut microbes can change drug effects because they can metabolize drugs and affect host physiological pathways. On the other hand, HF drugs can change the structure and function of the gut microbial community. This review also discusses how microbiome-related features may serve as biomarkers to support personalized treatment and how strategies such as dietary changes and microbiota-targeted therapies may improve clinical outcomes. Although evidence remains limited, and certain methods require further refinement, integrating microbiome insights into HF treatment may support more precise and individualized treatment strategies and help address current therapeutic limitations.

## 1 Introduction

Heart failure (HF) affects over 64 million people worldwide, and the number keeps rising because of population aging (McDonagh et al., 2021; Savarese et al., 2023). Moreover, mortality remains elevated. According to the National Heart Failure Audit (NHFA) 2023-2024, in-hospital mortality is 10.4%, and 30-day post-discharge mortality is 12.5% (National Heart Failure Audit, 2025). HF is the leading cause of hospitalization in adults older than 65, and it adds a large burden to healthcare systems (Liu et al., 2024; Savarese et al., 2023; Virani et al., 2021).

Pharmacotherapy remains central to the management of HF and has been shown to reduce morbidity and mortality. However, patient responses vary considerably, and many agents are associated with adverse effects, including arrhythmias, hypotension, renal dysfunction, and electrolyte disturbances (Heidenreich et al., 2022; Kaul, 2025). Although newer therapeutic classes, such as angiotensin receptor-neprilysin inhibitors (ARNIs), soluble guanylate cyclase (sGC) stimulators, and sodium-glucose cotransporter 2 (SGLT2) inhibitors, provide clinical benefit for some patients, substantial heterogeneity in efficacy and tolerability persists (Armstrong et al., 2020; Heidenreich et al., 2022; Solomon et al., 2019). Collectively, these observations suggest that traditional pharmacogenomics cannot explain all treatment differences.

Advances in high-throughput sequencing technologies have intensified interest in the gut microbiome, which is increasingly recognized as an “invisible organ” that exerts profound effects on host physiology and metabolism (Lynch and Pedersen, 2016; Niebauer et al., 1999; Sandek et al., 2007; Snelson et al., 2025; Zhao S et al., 2025). Patients with HF frequently exhibit gut dysbiosis, characterized by reduced microbial diversity, an expansion of pathobionts, and alterations in the Firmicutes-to-Bacteroidetes ratio (Chen A-T et al., 2023). Microbial metabolites have emerged as important mediators linking the gut microbiome to cardiovascular disease. Elevated levels of trimethylamine N-oxide (TMAO) are associated with accelerated atherosclerosis and adverse clinical outcomes, whereas reduced levels of short-chain fatty acids (SCFAs) may contribute to HF progression (Tang et al., 2017). Moreover, secondary bile acids have been shown to influence cardiac structure and metabolic remodeling (Bartolomaeus et al., 2019; Shen et al., 2025; Tang et al., 2019; Witkowski et al., 2020; Zhang et al., 2024).

Pharmacomicrobiomics has therefore become an important field that investigates the bidirectional interactions between the gut microbiome and drug responses (Hassan et al., 2021; Zhao et al., 2023). Microbial enzymes can change drug structures and bioavailability. These microbial transformations may influence key pharmacokinetic processes, including drug absorption, distribution, metabolism, and excretion (ADME) (Chen et al., 2022; Saha et al., 1983; Tuteja and Ferguson, 2019; Zhao et al., 2023). In addition to direct drug metabolism, the gut microbiome can modulate host drug-metabolizing enzymes and transporters, including cytochrome P450 (CYP450) enzymes, thereby further influencing drug pharmacokinetics and pharmacodynamics (Tuteja and Ferguson, 2019; Verdegaal and Goodman, 2024; Zimmermann et al., 2019). Conversely, medications themselves can reshape the gut microbial ecosystem. For example, commonly used drugs such as proton pump inhibitors and metformin have been shown to significantly alter microbial composition and metabolic activity, which may subsequently affect host physiology and treatment outcomes (Maier et al., 2018; Pant et al., 2023). Despite these advances, current pharmacokinetic assessments rarely incorporate the role of the gut microbiome in drug metabolism and response, highlighting an important knowledge gap in precision pharmacotherapy (Mak et al., 2022).

Despite growing recognition of the gut-heart axis and the substantial burden of HF, systematic understanding of the interactions between gut microbiota and HF pharmacotherapy remains limited. Existing studies have largely focused on microbial dysbiosis, metabolite associations, or disease pathogenesis, while the bidirectional interplay between microbiota and commonly used HF medications, which includes effects on drug metabolism, efficacy, and adverse events, has rarely been synthesized in a unified framework.

Given the limited efficacy of current therapies and the expanding understanding of microbiome-mediated modulation of drug responses, integrating microbiome insights into HF pharmacology has become increasingly important. Pharmacomicrobiomics provides a promising approach for elucidating interindividual variability in drug response and developing more precise, personalized treatment strategies. This review aims to summarize current evidence on gut microbiota-drug interactions in HF, highlight mechanistic insights and clinical implications, and outline key challenges and future directions in microbiome-informed precision medicine.

To identify relevant studies, literature searches were performed in PubMed, Web of Science, and Scopus databases up to January 2026. Search terms included combinations of “heart failure”, “gut microbiota”, “microbiome”, and “pharmacomicrobiomics”, together with specific drug classes such as “digoxin”, “ACE inhibitors”, “ARBs”, “SGLT2 inhibitors”, “β-blockers”, and “MRAs”.

## 2 Pharmacomicrobiology of heart failure therapeutics

**FIGURE 1:**
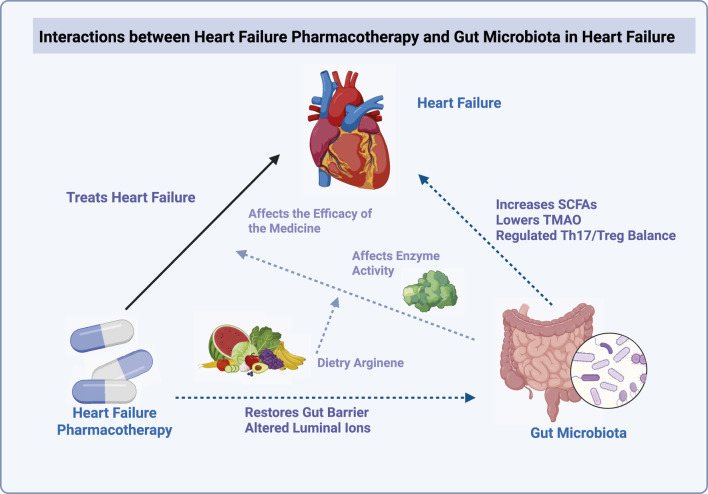
This diagram illustrates the complex interactions between HF pharmacotherapy and the gut microbiota. HF treatments may alter therapeutic efficacy by modifying the gut microbiota composition and its metabolic activities. Pharmacotherapy can influence gut microbiota through several mechanisms, such as enhancing or inhibiting specific enzymes, restoring gut barrier integrity, and altering luminal ion concentrations. Additionally, dietary factors, such as arginine, play a crucial role in modulating microbial activity. The gut microbiota itself can impact HF outcomes by increasing SCFA production, lowering TMAO, and regulating the balance of Th17/Treg cells, which are important for immune modulation.

The treatment of HF is shaped by two-way interactions between major drugs and the gut microbiome. This section reviews how key HF medications, including digoxin, SGLT2 inhibitors, renin-angiotensin-aldosterone system (RAAS) modulators, such as ACE inhibitors (ACEIs), angiotensin receptor blockers (ARBs) and ARNIs, diuretics, mineralocorticoid receptor antagonists (MRAs), β-blockers, and new therapeutic agents, interact with intestinal microbes. It explains how microbes can change drug efficacy and how drugs can change the structure and function of the gut microbial community (Figure 1; Table 1).

**TABLE 1:** Bidirectional interactions between heart failure drugs and the gut microbiota.

Drug class	Drug	Type of effect	Specific microbe/Metabolite	Direction	Mechanism
Cardiac Glycosides	Digoxin	Modulates Microbiota Composition	*Eggerthella lenta*	Cgr Activity↑	Inactivates digoxin, Lowering Active Plasma Levels (Koppel et al., 2018; Zimmermann et al., 2019)
			*Eggerthella lenta* (Arginine-regulated)	Cgr activity↓	Adequate Arginine Suppresses Cgr-Mediated Digoxin Inactivation (Ganamurali and Sabarathinam, 2025; Lu et al., 2014)
SGLT2 Inhibitors	Sotagliflozin	Modulates Microbiota Composition	e.g., *Alloprevotella*, Prevotellaceae UCG-001, NK3B31	Beneficial Taxa↑	Reduces Hypertrophy, Improves Ventricular Function (Liao et al., 2024; Steven et al., 2025)
	Dapagliflozin	Modulates Microbiota-Derived Metabolites	TMAO	TMAO↓	Ferroptosis-Related Genes; Reduces I/R Injury (Steven et al., 2025; Wang L et al., 2024)
	Empagliflozin/Dapagliflozin	Modulates Microbiota Composition and Microbiota-Derived Metabolites	SCFA-Producing Bacteria	SCFA-Producers and SCFAs↑	Anti-Inflammatory Signaling & Improved Gut-Barrier Integrity (Challa and Lewandowski, 2022; Chen H-C et al., 2023; Deng et al., 2022; Herat et al., 2020)
	SGLT2 Inhibitors	Modulates Microbiota Composition	Immune-Related Microbiota	-	Might Modulate SGK1/p-FoxO1/IL-23R Axis; Interacts with SCFA-regulated Th17/Treg Balance (Wang D et al., 2024)
ACE inhibitors/ARBs	Captopril	Modulates Microbiota Composition	Hypertension-Associated Dysbiotic Microbiota	Harmful Taxa↓	Restores Gut Barrier; Reduces Permeability & Fibrosis (Zimmermann et al., 2019)
	RAAS Inhibitors	Modulates Microbiota-Derived Metabolites	SCFAs	SCFAs↑	Regulate Renin *via* GPCRs and Olfr78^[17,62]^
			Bile-Acids	Altered BA Composition	Affects FXR/TGR5 to Modulate RAAS (Yu et al., 2024)
MRAs and Diuretics	Loop/Thiazide Diuretics	Modulates Microbiota-Derived Metabolites	Electrolyte-Driven Changes in Gut Environment	Altered Luminal Ions	Alter Luminal Na^+^/K^+^ and Osmotic Milieu and Shift Microbiota Metabolism and SCFA Production (Folz et al., 2019; Lucas et al., 2023; O’Donnell et al., 2023; Robles-Vera et al., 2020)
	MRAs	Modulates Microbiota Composition and Microbiota-Derived Metabolites	SCFAs and Bile Acids	SCFAs/Altered BA↑	Microbiota Influence Aldosterone Signaling and Systemic Inflammation (Pitt and Diez, 2024)
β-blockers	β-blockers	Modulates Microbiota Composition	-	-	Alter Gut-Sympathetic Crosstalk; Modify Host Sympathetic Tone *via* Microbiome-Mediated Pathways (Nagliya et al., 2024)
Iron Supplementation	Oral Iron	Modulates Microbiota Composition	Beneficial Commensals (SCFA-Producers)	Beneficial Taxa↑	Iron Deficiency-Induced Dysbiosis Impairs Iron Therapy Efficacy and Host Homeostasis (Asnicar et al., 2021; Das et al., 2020; Yao and Li, 2023; Y. Zhang, Wei, et al., 2025)

### 2.1 Digoxin and gut microbiome interactions as a Paradigm of microbe-driven pharmacokinetics

Digoxin is a cardiac glycoside widely used in HF and works mainly by inhibiting Na^+^/K^+^-ATPase to improve myocardial contractility. Some patients benefit from it, but others show weak responses or develop toxicity, such as arrhythmias (Crane et al., 2024; Heidenreich et al., 2022; Kaul, 2025; Patocka et al., 2020).

Pharmacomicrobiomics has provided important insights into this variability. Early studies demonstrated that some gut bacteria, especially *Eggerthella lenta*, can express reductase enzymes that inactivate digoxin. These enzymes lower digoxin levels and reduce its efficacy (Javdan et al., 2020). Antibiotics may increase digoxin absorption because they reduce early microbial degradation in the gut (Crane et al., 2024). Later studies found that this pathway varies greatly between individuals because *E. lenta* may or may not carry an operon that encodes cardiac glycoside reductase (Cgr) operon (Koppel et al., 2018; Zimmermann et al., 2019). This is one of the most well-known examples of drug-microbiota interactions and helps explain patient-specific differences in drug response. More recent findings show that digoxin can also alter gut microbiome composition and may increase the risk of intestinal *Salmonella* infection, so the interaction is two-way (Kumar et al., 2025). Other studies have shown that the dehydrogenase activity of *E. lenta* depends on dietary arginine, and adequate arginine intake can reduce digoxin inactivation. This points to a diet-microbiota-drug interaction (Ganamurali and Sabarathinam, 2025; Lu et al., 2014).

Clinical observational studies report that digoxin use is linked with increased all-cause mortality and sudden cardiac death in patients with atrial fibrillation or HF(Angraal et al., 2019; Volgman et al., 2022; Hack et al., 2025). These observations may be affected by factors such as disease severity, comorbidities, and dose monitoring. But they may also be partly due to differences in gut microbial composition and metabolic activity. Microbial features may therefore be important for determining both digoxin efficacy and toxicity.

Current evidence on digoxin-microbiome interactions mostly comes from case reports and small studies, so large prospective trials are still missing. Future work should focus on two main goals. One goal is to test whether microbial profiles can be used as biomarkers to predict digoxin response and toxicity. The other goal is to test whether dietary changes or targeted inhibitors of microbial enzymes can improve the safety and effectiveness of digoxin therapy. If these gaps are addressed, digoxin may gain renewed value in precision pharmacotherapy.

### 2.2 Gut microbiota mediation of SGLT2 inhibitors in cardioprotective effects beyond glycemic control

SGLT2 inhibitors have expanded far beyond glycemic control. Clinical studies show that SGLT2 inhibitors improve outcomes in patients with HF, and this is true in those with or without diabetes and in different HF phenotypes. These benefits may come from diuresis, higher myocardial ATP availability, and better vascular function (Cai et al., 2024; Cowie and Fisher, 2020; Dasari et al., 2023; Lee et al., 2025; Rådholm et al., 2018).

Animal studies suggest that some of these cardioprotective effects may involve the gut microbiome. Experimental models show that SGLT2 inhibitors can alter gut microbial diversity (Billing et al., 2024). In mouse models of myocardial infarction, sotagliflozin changes microbial communities such as *Alloprevotella*, Prevotellaceae *UCG-001*, and Prevotellaceae *NK3B31*, and this is linked to reduced cardiac hypertrophy and apoptosis and better cardiac function (Liao et al., 2024; Steven et al., 2025). Similarly, in diabetic rats with myocardial ischemia-reperfusion injury, dapagliflozin lowers trimethylamine N-oxide (TMAO), which is a microbial metabolite associated with adverse remodeling, because it affects ALB, PPARG, and HMOX1 pathways involved in cardiomyocyte ferroptosis. This results in less injury and better cardiac function (Wang L et al., 2024). Moreover, short-chain fatty acids (SCFAs) may also play a role because they have anti-inflammatory and immunomodulatory effects, they strengthen the gut barrier, and they influence cardiac contractility. In a randomized two-arm clinical trial, empagliflozin increased SCFA-producing bacteria, and dapagliflozin raised SCFA levels in mouse models of neurogenic hypertension. These changes may help improve HF through a microbiota-metabolite pathway (Challa and Lewandowski, 2022; Chen H-C et al., 2023; Deng et al., 2022; Herat et al., 2020). Other findings suggest that SGLT2 inhibitors may influence immune responses because they change local sodium concentrations. This affects the SGK1/p-FoxO1/IL-23R axis and helps restore the Th17/Treg balance (Wang H et al., 2024). Microbial metabolites such as SCFAs can also affect the Th17/Treg axis and influence cardiac inflammation and fibrosis (Bai et al., 2023; Bartolomaeus et al., 2019; Xia et al., 2024). These findings point to a possible microbiota-immune-heart pathway, but this idea still needs more evidence. In addition, a two-sample, two-step Mendelian randomization study shows that SGLT2 inhibitors can change bile acid metabolic pathways, which depend on gut microbial activity. This provides another clue to the drug-microbiota-host interaction (Xu et al., 2022).

Large randomized controlled trials have not yet provided direct causal evidence that the gut microbiome mediates the cardioprotective effects of SGLT2 inhibitors. Current results mainly come from animal studies and small metabolomic analyses; therefore the extent of microbial involvement in clinical benefits is still unknown. Future studies should employ multi-omics approaches, such as metagenomics and metabolomics, in combination with longitudinal clinical data to determine whether SGLT2 inhibitors exert their effects partly through microbiome-related pathways. Such work may also facilitate the identification of microbial markers that could guide individualized SGLT2 inhibitor therapy in HF.

### 2.3 Bidirectional crosstalk between RAAS inhibitors and gut microbiome shapes therapeutic response

ACEIs, ARBs, and ARNIs are key drugs in current HF treatment (Heidenreich et al., 2022). Many randomized controlled trials and meta-analyses show that these drugs reduce HF-related hospitalizations and all-cause mortality (Lin et al., 2022; Mou et al., 2024; Tsigkas et al., 2022; Vaduganathan et al., 2020).

RAAS activity depends on host genetics and endocrine signals, and it also depends on the gut microbiome (Gabriel and Ferguson, 2023). Experimental studies demonstrate that microbial metabolites such as SCFAs and propionate can activate G protein-coupled receptors (GPCRs) and change olfactory receptor 78 (Olfr78) expression in the juxtaglomerular apparatus. These changes affect renin release, blood pressure, and inflammation (Bartolomaeus et al., 2019; Gotoh and Shibata, 2023). In addition, certain gut microbes can also alter bile acid metabolism and affect the Farnesoid X receptor/Takeda G protein-coupled receptor 5 (FXR/TGR5) pathway, so they can influence RAAS activity in spontaneously hypertensive rat models (Yu et al., 2024). Because ACEIs, ARBs, and ARNIs act on this pathway, they may also interact with the gut microbiome. For example, captopril can reverse hypertension-related dysbiosis by lowering intestinal permeability and fibrosis and by restoring villus structure and mucosal integrity in spontaneously hypertensive and chronic angiotensin II infusion rat models (Tuteja and Ferguson, 2019). Clinical studies indicate that patients with gut dysbiosis may have more variable responses to ACEIs and ARBs, and this suggests that microbiota composition may be an important factor in drug-response differences (Kumar et al., 2025; Yang et al., 2022).

Human evidence for these interactions remains limited. Therefore future research should identify microbial taxa or metabolites linked to treatment responses to RAAS inhibitors, and combine multi-omics data with clinical outcomes to show how microbial metabolism affects drug efficacy and adverse events. These efforts may support microbiome-informed treatment strategies and help develop more precise HF therapy.

### 2.4 Microbiome linked mechanisms underlying the actions of diuretics and mineralocorticoid receptor antagonists

MRAs are widely used in the treatment of HF (Heidenreich et al., 2022). Their main therapeutic effects come from regulating fluid balance and neurohormonal activity, and this helps reduce cardiac workload (Chang et al., 2024; Cosentino et al., 2023; Pitt and Byrd, 2021).

Recent studies suggest that the gut microbiome may affect the efficacy and safety of these drugs. Chronic diuretic use often leads to electrolyte imbalances such as hypokalemia and hyponatremia, and these shifts can change the intestinal environment and microbial stability. Changes in sodium and potassium levels can affect bacterial osmotic control and growth, thereby reshaping microbial composition and modifies SCFA levels. SCFAs can then influence host immune responses (Folz et al., 2019; Lucas et al., 2023; O’Donnell et al., 2023; Pitt and Diez, 2024; Robles-Vera et al., 2020). Spironolactone can also reduce sympathetic activity in the gut, alter microbial composition, and influence systemic aldosterone metabolism in spontaneously hypertensive rat models (González-Correa et al., 2023). When intestinal microbiota are inhibited, plasma and urinary aldosterone levels increase (Moore et al., 2024). MRAs lower systemic inflammation and fibrosis, and some of these effects may depend on microbial metabolites such as SCFAs and bile acid derivatives because they act through immunomodulatory pathways (Pitt and Diez, 2024). Animal studies further indicate that excess aldosterone activity increases intestinal permeability and causes dysbiosis, and MRAs can partially reverse these changes (González-Correa et al., 2023).

Current evidence provides useful mechanistic clues, but direct clinical studies that assess how diuretics and MRAs affect the gut microbiome, or whether microbiome features predict treatment response or adverse events, are still limited. Therefore future work should focus on evaluating microbiome changes during therapy and linking them with HF outcomes, clarifying how electrolytes shape the gut environment and microbial behavior, and identifying microbial biomarkers related to hyperkalemia or hypokalemia risk to support safer and more personalized treatment strategies.

### 2.5 β-blockers and the sympathetic gut axis in host microbe drug interactions

β-blockers are a key component of chronic HF management (Paolillo et al., 2021). They improve cardiac function and survival because they inhibit sympathetic nervous system activity, reduce heart rate, and lower myocardial oxygen demand (Heidenreich et al., 2022).

Recent studies suggest the existence of a potential “sympathetic-gut axis.” Sympathetic activation may affect not only cardiac and vascular function but also intestinal blood flow, motility, and secretion, and these changes may reshape the gut environment and alter microbial composition and activity (Santisteban et al., 2017; Wang et al., 2021). Microbial metabolites, especially SCFAs, may also act on the vagus nerve or peripheral receptors and influence sympathetic tone and inflammation. For example, propionate and acetate may activate G protein-coupled receptors (GPR41/43) on vascular smooth muscle, and this may modulate vasodilation and interact with hemodynamic effects produced by β-blockers (Nagliya et al., 2024). These observations suggest that the effects of β-blockers may be partly shaped by the gut microbiome.

Direct evidence linking β-blockers to changes in the gut microbiota remains limited. Clinical observations have reported substantial interindividual variability in heart rate control, adverse events, and overall treatment benefit, and these differences may relate to microbial diversity and metabolite patterns (Brocker et al., 2020; Jackson et al., 2018; Lin et al., 2021). But this link is still speculative and needs stronger mechanistic support. Therefore, future research should employ multi-omics approaches to identify relationships between β-blocker responses and microbial or metabolic features, examine the role of the sympathetic-gut axis in cardioprotection, and test whether specific microbial biomarkers can predict β-blocker responsiveness to support more personalized HF therapy.

### 2.6 Emerging heart failure therapeutics and potential interactions with gut microbiome

Beyond conventional HF medications, growing attention has been given to how new therapeutic agents interact with the gut microbiome. One important example is iron supplementation, which exhibits bidirectional interactions with intestinal microbial communities. Iron deficiency is a common condition in HF patients, and it can change the intestinal environment and shift microbial composition (Fang et al., 2023; Qi et al., 2020; Yao and Li, 2023). Gut microbes can also affect iron absorption, metabolism, and bioavailability because they regulate transcription factors such as hypoxia-inducible factor 2α (HIF-2α) or modulate ferroptosis pathways, and this may influence systemic iron balance and HF progression (Asnicar et al., 2021; Das et al., 2020; Yao and Li, 2023; Zhang, Wei, et al., 2025). Clinical trials, including FAIR-HF and CONFIRM-HF trials, have demonstrated that intravenous iron therapy can improve symptoms and quality of life in HF patients, but the mechanisms remain unclear, suggesting that the gut microbiota may act as an unrecognized regulatory factor (Lakhal-Littleton and Cleland, 2024).

New therapeutic agents such as vericiguat, a soluble guanylate cyclase (sGC) stimulator, and omecamtiv mecarbil, a cardiac myosin activator, also show clear clinical benefits in HF management (Armstrong et al., 2020; Teerlink et al., 2021). But it is not known whether these drugs interact with the gut microbiome, and this represents a major evidence gap. Because their mechanisms involve the nitric oxide (NO) signaling pathway and myocardial energy metabolism, future studies should examine possible crosstalk with microbial metabolites such as NO-related molecules and SCFAs. Such work may reveal new mechanisms and support the development of microbiome-informed precision medicine for HF.

This diagram illustrates the complex interactions between HF pharmacotherapy and the gut microbiota. HF treatments may alter therapeutic efficacy by modifying the gut microbiota composition and its metabolic activities. Pharmacotherapy can influence gut microbiota through several mechanisms, such as enhancing or inhibiting specific enzymes, restoring gut barrier integrity, and altering luminal ion concentrations. Additionally, dietary factors, such as arginine, play a crucial role in modulating microbial activity. The gut microbiota itself can impact HF outcomes by increasing SCFA production, lowering TMAO, and regulating the balance of Th17/Treg cells, which are important for immune modulation.

## 3 Translating experimental insights into clinical applications

**FIGURE 2:**
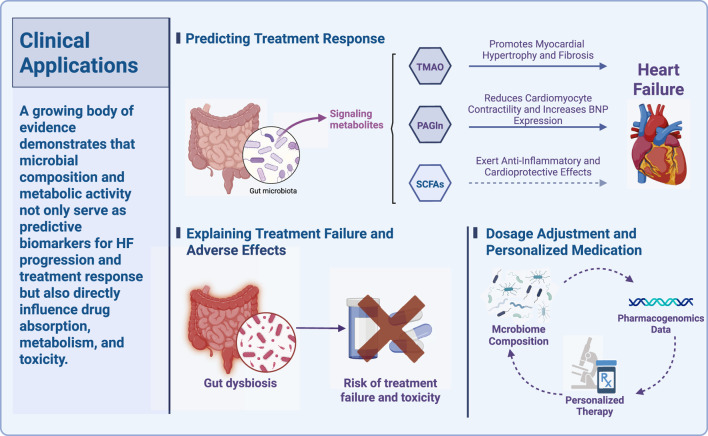
This diagram highlights the clinical relevance of gut microbiome composition and its metabolites in the management of HF. Microbial biomarkers, including signaling metabolites such as TMAO, PAGln, and SCFAs, play pivotal roles in predicting treatment responses by influencing myocardial hypertrophy, cardiomyocyte contractility, and BNP expression. Additionally, gut microbiota composition and dysbiosis are linked to treatment failure and adverse drug effects, suggesting their potential as biomarkers for identifying individuals at risk of poor therapeutic outcomes and toxicity. The integration of microbiome composition with pharmacogenomics data further supports personalized therapy and dosage adjustments, advancing individualized management strategies for HF patients.

Building on established drug-microbiota interactions, the gut microbiome is now viewed as an important factor for clinical translation in HF. This section examines its role in three main areas. First, it can provide predictive biomarkers for treatment response and clinical outcomes. Second, it can act as a mediator of failed treatment and adverse drug reactions. Third, it can also serve as a variable that should be included in personalized dosing and therapeutic modeling, so it may help move from mechanistic insight to precision therapy (Figure 2).

### 3.1 Microbiome-derived biomarkers for predicting therapeutic response and clinical outcomes

Identifying reliable biomarkers that can predict drug response and clinical outcomes is essential for personalized therapy in HF. In recent years, the gut microbiome and its metabolites have been viewed as promising indicators of treatment efficacy and prognosis.

The gut microbial metabolite TMAO is strongly associated with adverse cardiovascular outcomes. Higher TMAO levels can promote myocardial hypertrophy and fibrosis, increase systemic inflammation, and impair mitochondrial function, thereby exacerbating HF progression. Patients with high baseline TMAO often respond poorly to standard therapies and exhibit higher risks of rehospitalization and mortality (Suzuki et al., 2019; Zhang Y et al., 2021).

Phenylacetylglutamine (PAGln), another gut microbial metabolite derived from phenylalanine metabolism, may also contribute to HF-related pathophysiological changes. It has been shown to reduce sarcomere contraction and increase brain natriuretic peptide (BNP) gene expression in both cultured cells and HF mouse models. In addition, PAGln levels also correlate with atherosclerosis severity and cardiovascular event risk, suggesting its potential utility in predicting HF risk and treatment response (Romano et al., 2023; Tang et al., 2024; Zong et al., 2022; Yi et al., 2024).

Protective metabolites may signal better outcomes. SCFAs produced through microbial fermentation help maintain gut barrier integrity and reduce inflammation. Reduced SCFA levels have been linked with more severe HF and higher systemic inflammation (Hu et al., 2022).

Even though these findings are encouraging, most microbial biomarkers are still not ready for clinical application. Standardized testing methods and diagnostic cutoffs are lacking, and only a limited number of studies have validated these markers in large and diverse HF populations. Future studies should leverage existing HF cohorts and clinical biobanks and combine metagenomic, metabolomic, and clinical data to test whether microbiome-based markers add value beyond traditional biomarkers. AI-based models may also enhance risk prediction and support real-time, microbiome-guided treatment decisions in HF.

### 3.2 Microbiome-mediated mechanisms of treatment failure and adverse drug reactions

Variability in therapeutic efficacy and the occurrence of adverse drug reactions (ADRs) in HF treatment are often linked to factors such as medication indication, disease heterogeneity, comorbidities, and inadequate dose monitoring. However, growing evidence suggests that differences in gut microbiome composition and function may also play an important role. The gut microbiota can affect drug efficacy and safety because it can directly metabolize drugs, induce systemic inflammation, and produce metabolites that may harm the heart or kidneys.

Clinical and preclinical studies show that dysbiosis, which includes reduced microbial diversity, expansion of pathogenic taxa, and accumulation of specific metabolites, is closely linked with treatment failure and higher toxicity risk. TMAO is the most widely studied risk metabolite. High TMAO levels are associated with poor HF prognosis and can promote platelet activation (Duttaroy, 2021), induce myocardial fibrosis (Khalil et al., 2017), and facilitate arrhythmogenesis (Fan et al., 2023; Yu et al., 2018). These effects may help explain why some patients respond poorly to standard therapy. Secondary bile acids (such as deoxycholic acid) and indole derivatives (such as indoxyl sulfate) can also activate inflammatory pathways, increase oxidative stress, and impair endothelial function, so they worsen cardiac remodeling and renal injury and raise the risk of drug-related adverse effects (Du et al., 2020; Frazier et al., 2022; Jovanovich et al., 2018; Qi et al., 2025; Wakamatsu et al., 2024; Zhang, Han, et al., 2025).

These findings suggest that microbiome and metabolome profiling may help identify HF patients who are at high risk for poor treatment response or toxicity, so clinicians can apply more individualized pharmacologic strategies. However, most evidence comes from observational studies and animal experiments, and causal links still need confirmation through interventional trials. Future research should focus on targeted microbiota modulation, including probiotic, prebiotic, and dietary interventions, and integrate these with multi-omics approaches and AI-based prediction models. Such work may show whether modifying specific microbial functions or metabolic pathways can improve therapeutic efficacy and reduce drug toxicity, and it may also promote the development of precision pharmacovigilance in HF management.

### 3.3 Integrating microbiome insights into personalized dosing and therapeutic modeling

Marked interindividual variability exists in therapeutic responses to HF treatments. These differences are usually linked to genetic background, hepatic and renal function, and comorbidities. However, growing evidence shows that the structure and function of the gut microbiome also play an important role in determining drug efficacy. The microbiome can alter drug biotransformation, activation, and degradation, resulting in substantial interindividual differences in drug exposure and therapeutic response even when they receive the same dose (Anwar et al., 2025; Tsunoda et al., 2021).

Digoxin is a classical example. Studies have shown that some patients harbor *Eggerthella lenta* in the gut, and this bacterium can reduce and inactivate digoxin. As a result, plasma concentrations fall below therapeutic levels and drug efficacy decreases (Trepka et al., 2025; Tsunoda et al., 2021). This suggests that patients with these digoxin-reducing microbes may need dose adjustments or tailored administration strategies. Similar microbiome-dependent effects may occur with other HF medications, and microbial profiling may be a key step for explaining dose-response variability and for improving patient stratification and dosage optimization.

Pharmacogenomics has advanced individualized treatment but cannot fully explain the range of drug-response differences. Integrating microbiome data with host genomic information may create a more complete predictive system (Cai et al., 2023). For example, in patients treated with RAAS inhibitors, SGLT2 inhibitors, or β-blockers, analyzing both drug-metabolizing gene polymorphisms (such as CYP450 variants) and microbial metabolic capacity may better predict drug efficacy and adverse reaction risk, so clinicians can refine dosing and treatment choices (Weersma et al., 2020; Zimmermann et al., 2019).

Reaching this goal requires several advances. These include adding microbial metabolic rates and activity parameters into physiologically based pharmacokinetic (PBPK) and pharmacokinetic (PK) models to support drug development and dose prediction (Zhuang and Lu, 2016), creating standardized microbiome diagnostic tools, and building interpretable machine-learning models. These models must also be validated using real-world evidence and prospective clinical trials to evaluate their impact on outcomes and cost-effectiveness. Together, these efforts may help bring microbiome-informed personalized HF therapy into practical application.

This diagram highlights the clinical relevance of gut microbiome composition and its metabolites in the management of HF. Microbial biomarkers, including signaling metabolites such as TMAO, PAGln, and SCFAs, play pivotal roles in predicting treatment responses by influencing myocardial hypertrophy, cardiomyocyte contractility, and BNP expression. Additionally, gut microbiota composition and dysbiosis are linked to treatment failure and adverse drug effects, suggesting their potential as biomarkers for identifying individuals at risk of poor therapeutic outcomes and toxicity. The integration of microbiome composition with pharmacogenomics data further supports personalized therapy and dosage adjustments, advancing individualized management strategies for HF patients.

## 4 Microbiome-based therapeutic strategies in heart failure

Translating microbiome science into clinical practice requires several types of interventions. This section reviews microbiome-targeted strategies for HF, including dietary changes, adjunctive therapies such as probiotics and fecal microbiota transplantation (FMT), and new drugs that target microbial pathways. Each strategy is described based on its mechanism, its potential for clinical use, and the main challenges that need to be addressed (Table 2).

**TABLE 2:** Translational strategies targeting the gut-heart axis in heart failure.

Intervention	Type of effect	Microbe/Metabolite	Direction	Mechanistic phrase
High-Fiber Diets/Fermentable Fiber	Modulates Microbiota Composition and Microbiota-Derived Metabolites	SCFA-Producers (e.g., *Akkermansia*, *Lactobacillus*, *Faecalibacterium*)	Beneficial Taxa↑ and SCFAs↑	Barrier Integrity enhancement, Systemic Inflammation Reduction, and Cardiac Function Improvement (Avellaneda-Franco et al., 2024; Deehan et al., 2024; Pakhomov and Baugh, 2021; Pereira et al., 2024; Yin et al., 2025; Zuo et al., 2022)
Polyphenol-Rich Diets	Modulates Microbiota Composition	Polyphenol-metabolizing commensals↑ (e.g., *Gordonibacter*, *Bifidobacterium*, *Akkermansia*)	Beneficial Taxa↑	Antioxidant, Anti-Inflammatory, and Microbiota-Remodeling Effects (Alqudah and Claesen, 2024; Bianchi et al., 2022; El-Saadony et al., 2024; Molinari et al., 2022; Petersen and Mansell, 2025; Xu et al., 2021; Zhang et al., 2018)
Ketogenic Diet	Modulates Microbiota Composition	Beneficial commensals↓ (e.g., *Lactobacillus*, *Bifidobacterium*)	Beneficial Taxa↑	Impaired Mitochondrial Biogenesis and Promoted Fibrosis/Apoptosis (Xu et al., 2021; Yin et al., 2025)
Probiotics/Prebiotics/Synbiotics	Modulates Microbiota Composition and Microbiota-Derived Metabolites	Enrichment of Administered Strains (e.g., *Lactobacillus*, *Bifidobacterium*, *C. butyricum*)	Administered Taxa↑ SCFAs ↑ and TMAO↓	Attenuated Inflammation and Myocardial Remodeling (Karmazyn and Gan, 2023; Kaye et al., 2020)
Fecal Microbiota Transplantation (FMT)	Modulates Microbiota Composition	Broad Community Restructuring	Beneficial Taxa↑	Reduced BP, Oxidative Stress, and Inflammation (Luo et al., 2023; Luqman et al., 2024)
Engineered Live Biotherapeutics	Modulates Microbiota Composition	Engineered Strains Colonize Gut (e.g., NAPE-expressing strains)	Engineered Taxa↑	Modulated Host Metabolism and Atherosclerotic/Cardiometabolic Indices (Brödel et al., 2024; May-Zhang et al., 2019)
Microbial Enzyme Inhibitors	Modulates Microbiota-Derived Metabolites	TMA-Producing Taxa or Enzymes (e.g., DMB/FMO3 inhibitors)	TMAO↓	Attenuated Cardiac Remodeling (Lv et al., 2022; Yin et al., 2025)

### 4.1 Dietary modulation of the gut microbiome in heart failure

**FIGURE 3:**
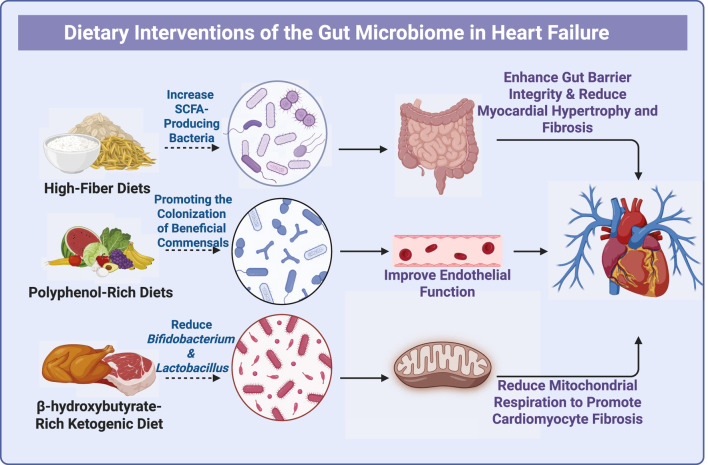
This diagram illustrates the impact of dietary interventions on the gut microbiome and their subsequent effects on HF. High-fiber diets are shown to increase SCFA-producing bacteria, which enhance gut barrier integrity and reduce myocardial hypertrophy and fibrosis. Polyphenol-rich diets promote the colonization of beneficial commensals, improving endothelial function and contributing to overall cardiovascular health. In contrast, a β-hydroxybutyrate-rich ketogenic diet decreases beneficial microbes such as Bifidobacterium and Lactobacillus, while potentially reducing mitochondrial respiration and promoting cardiomyocyte fibrosis. These dietary approaches highlight the importance of gut microbiota modulation as a therapeutic strategy for HF management

Diet is one of the most important environmental factors that shape the gut microbiome (Losno et al., 2021; Yang et al., 2021), and it may serve as a useful adjunctive approach for HF management. Evidence shows that specific dietary patterns can change microbial composition and metabolic activity, and these changes may affect HF progression and outcomes (Fadhillah et al., 2025) (Figure 3).

High-fiber diets exhibit clear cardioprotective effects in hypertensive mouse models. They can lower blood pressure (Avellaneda-Franco et al., 2024), enhance cardiac function, and reduce myocardial hypertrophy and fibrosis (Yin et al., 2025). These benefits appear because high-fiber intake increases SCFA-producing bacteria such as *Akkermansia muciniphila* and *Lactobacillus* spp. (Pakhomov and Baugh, 2021), enhances intestinal barrier integrity (Pereira et al., 2024), lowers inflammation (Zuo et al., 2022), and improves host energy metabolism (Deehan et al., 2024). Human studies further indicate that low-fiber intake is linked to higher HF risk and poorer outcomes, and that adequate fermentable fiber may raise SCFA levels, reduce systemic inflammation, and provide cardioprotection (Pakhomov and Baugh, 2021). In contrast, ketogenic diets enriched in β-hydroxybutyrate have been shown to reduce *Lactobacillus* and *Bifidobacterium* abundance in rat models, and this can increase mitochondrial stress, cardiomyocyte apoptosis, and fibrosis (Xu et al., 2021; Yin et al., 2025). These findings highlight that different dietary patterns can have very different effects on the gut-heart axis.

Polyphenol-rich diets, which are common in fruits, vegetables, and whole grains (El-Saadony et al., 2024), can also reshape the gut microbiota. Such diets have been shown to increase commensal colonization (Molinari et al., 2022; Petersen and Mansell, 2025), prevent pathogen overgrowth (Bianchi et al., 2022), and lower oxidative stress and intestinal permeability (Alqudah and Claesen, 2024). Small clinical studies further suggest that polyphenol supplementation can reduce inflammation and improve endothelial function (Alqudah and Claesen, 2024; Zhang et al., 2018). Direct evidence in HF remains limited, but current data support the relevance of the diet-microbiome-heart axis.

Future studies should include large, long-term randomized controlled trials to test how dietary patterns such as the Mediterranean diet or customized high-fiber or polyphenol-rich plans affect the gut microbiome, metabolic phenotypes, and clinical outcomes such as rehospitalization and mortality in HF patients. Researchers should also consider patient adherence and cultural dietary differences. Using metabolomic and microbiomic data to identify responders, and testing how diet works together with HF drugs such as SGLT2 inhibitors and ARNIs, will be important for advancing dietary microbiome-based precision therapy.

This diagram illustrates the impact of dietary interventions on the gut microbiome and their subsequent effects on HF. High-fiber diets are shown to increase SCFA-producing bacteria, which enhance gut barrier integrity and reduce myocardial hypertrophy and fibrosis. Polyphenol-rich diets promote the colonization of beneficial commensals, improving endothelial function and contributing to overall cardiovascular health. In contrast, a β-hydroxybutyrate-rich ketogenic diet decreases beneficial microbes such as Bifidobacterium and *Lactobacillus*, while potentially reducing mitochondrial respiration and promoting cardiomyocyte fibrosis. These dietary approaches highlight the importance of gut microbiota modulation as a therapeutic strategy for HF management.

### 4.2 Microbiota-targeted adjunctive therapies in heart failure

Direct modulation of the gut microbiome is another promising translational strategy for managing HF. Current methods include probiotics, prebiotics, FMT, and supplementation with microbial metabolites (Mamic et al., 2021). Studies show that probiotics can lower proinflammatory cytokines and reduce TMAO production, and this can lessen cardiac hypertrophy and improve left ventricular function (Karmazyn and Gan, 2023). Prebiotics can support the growth of beneficial microbes, and this increases SCFA production and activates GPR43 and 109A signaling, which leads to cardioprotective effects in hypertensive mouse models (Kaye et al., 2020). FMT has been shown to reduce systolic blood pressure, improve vascular function, and decrease oxidative stress and inflammation in the vasculature (Luo et al., 2023; Luqman et al., 2024). In addition, animal studies in spontaneously hypertensive rats suggest that restoring the microbiota through FMT may also improve drug response and may serve as a new approach for reshaping the gut microbial ecosystem in HF(Yun et al., 2025).

Microbial metabolites appear to mediate many of these effects. SCFAs can improve cardiac function by supporting immune regulation and energy metabolism (Hu et al., 2022). Bile acid signaling pathways, including FXR and TGR5, also regulate myocardial energy use and inflammation, and they influence HF development and progression (Fleishman & Kumar, 2024; Wang H et al., 2024). These findings suggest that metabolite-based treatments, such as SCFA supplementation or modulation of bile acid pathways, may serve as new therapeutic options for HF.

Engineered gut bacteria represent another emerging direction. These bacteria can deliver defined therapeutic compounds in a continuous and targeted manner. In low-density lipoprotein receptor deficient mouse models, engineered strains expressing N-acyl-phosphatidylethanolamine (NAPE) can be added to the gut microbiota and slow the progression of atherosclerosis and related cardiometabolic diseases (May-Zhang et al., 2019). In a separate approach, bacteriophage-derived particles have been used to deliver base editors for *in situ* genome modification of *Escherichia coli* inside the mouse gut, showing that microbial genome editing is possible *in vivo* (Brödel et al., 2024). Given the close connection between the gut and the heart, microbiome engineering may eventually support the development of precision therapies for HF.

Even with these promising findings, most evidence still comes from animal studies and early-phase clinical investigations. Large randomized controlled trials (RCTs) and real-world data remain limited. Important questions remain about long-term safety, stability of microbial engraftment, effectiveness of engineered strains, and optimal formulations and dosing. Future studies should include multicenter RCTs in HF patients to test the safety and efficacy of microbiota-targeted therapies. They should also examine how these therapies may work together with standard HF drugs (e.g., SGLT2 inhibitors, ARNIs) and include microbiome profiling to guide personalized treatment. These efforts will be important for moving microbiota-based therapies from experimental concepts into precise clinical applications.

### 4.3 Development of novel microbiome-related drugs for heart failure

With growing recognition of the role of microbial metabolites in HF pathogenesis, the development of drugs that target microbial metabolic pathways has become a major research focus. One key area involves TMAO inhibition. In animal models, 3,3-dimethyl-1-butanol (DMB) suppresses TMAO synthesis and improves post-myocardial infarction remodeling, fibrosis, and inflammation (Yin et al., 2025). Inhibitors of flavin-containing monooxygenase 3 (FMO3), a central enzyme in trimethylamine (TMA) formation, are also under investigation, and they highlight the therapeutic potential of modulating TMAO generation or metabolism in HF(Lv et al., 2022).

Beyond the TMAO pathway, other targets include microbial enzymes involved in bile acid metabolism and bioactive metabolite analogs such as SCFA receptor agonists or bile acid receptor derivatives (Park et al., 2022; Yin et al., 2021; Zhao M et al., 2025). These strategies allow more precise modulation of host-microbe interactions, and they may reduce variability in drug response and the risk of adverse events.

Most candidate compounds remain in preclinical development, so translation into human studies is still required. Future drug development should include early evaluation of microbiome-drug interactions because these interactions can shape pharmacokinetics and pharmacodynamics. Integration of multi-omics technologies and AI-assisted target screening may further improve drug design, enhance safety and efficacy, and accelerate clinical translation of microbiome-targeted therapeutics for HF.

### 4.4 Personalized microecological interventions in heart failure management

Advances in microbiome science and precision medicine are enabling personalized microecological interventions as a new direction in HF management. Differences in population background, age, sex, comorbidities (such as renal dysfunction or diabetes), and polypharmacy (such as proton pump inhibitors, Aspirin, or Atorvastatin) can all shape gut microbiota composition and function in HF patients (Forslund et al., 2021; Lai et al., 2025; Ma et al., 2024; Peters et al., 2022; Wilson et al., 2020; Zhu et al., 2024; Zhuang et al., 2019).

Personalized interventions aim to tailor microbiota-targeted treatment based on an individual’s microbial composition, metabolic phenotype, and functional profile so that therapy becomes more effective and safer. Possible future applications include customized probiotic or prebiotic formulations for specific HF phenotypes. For example, patients with inflammatory activation may benefit from SCFA-producing anti-inflammatory strains, and patients with reduced microbial diversity may receive targeted prebiotics to promote the growth of key commensal taxa. Metabolomics-guided approaches could also support the design of metabolite analogues or antagonists, such as TMAO inhibitors or SCFA receptor agonists, to directly modulate pathways linked to HF progression.

However, several challenges remain. These include establishing robust microbiota classification systems; developing rapid and low-cost diagnostic tools; confirming the effectiveness and long-term safety of customized formulations across diverse populations; and addressing issues related to adherence, manufacturing quality, and regulatory standards. Integration of multi-omics profiling with AI-based predictive modeling may help identify meaningful microbial biomarkers and forecast individual responses to specific interventions. Combining these tools with real-world data and multicenter clinical validation may shift HF care from a “one-size-fits-all” approach to a dynamic and precision-tailored microecological treatment model. Although still in an early stage, this strategy holds significant promise for advancing personalized HF management.

## 5 Challenges and future roadmaps in pharmacomicrobiomics of heart failure

**FIGURE 4:**
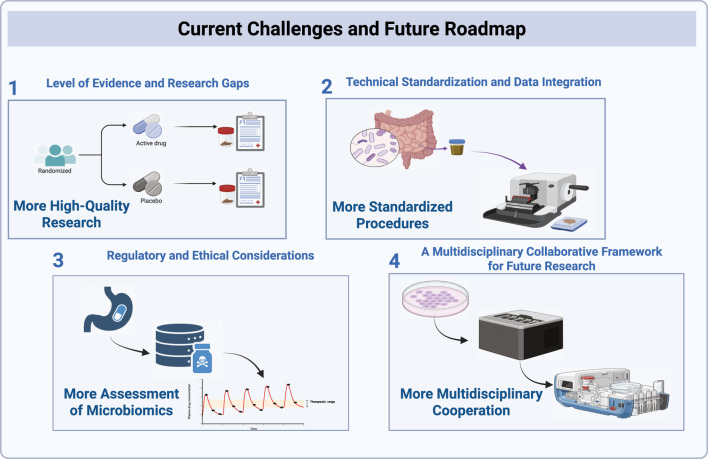
This diagram outlines the key challenges and future directions in microbiome research related to HF. The first challenge emphasizes the need for more high-quality randomized studies to address research gaps and strengthen the evidence base. The second challenge focuses on the technical standardization and integration of microbiome data, highlighting the need for standardized procedures to ensure consistent and reproducible results. The third challenge involves the assessment of microbiome research, with an emphasis on regulatory and ethical considerations related to gut microbiome interventions. Finally, the fourth challenge underscores the importance of multidisciplinary cooperation, with a call for greater collaboration across fields to accelerate microbiome-based therapies in HF management.

Pharmacomicrobiomics offers a powerful framework for understanding therapeutic variability in HF, but its path to clinical adoption remains challenging. Evidence gaps, methodological inconsistencies, and unresolved regulatory and ethical issues still limit translation. This concluding section summarizes these barriers and outlines a roadmap for the field, emphasizing the need for methodological standardization, multi-omics and AI integration, rigorous clinical validation, and interdisciplinary collaboration to move microbiome-guided therapy from promise to practice (Figure 4).

### 5.1 Evidence limitations and research gaps in current studies

Research on pharmacomicrobiomics in HF is still at an early stage, and current evidence remains heterogeneous. Many studies have small sample sizes, short follow-up periods, and observational designs, so causal inference is limited. In addition, experimental findings rely heavily on animal models, and interspecies differences restrict translational relevance.

To strengthen causal interpretation, future work should include large, multicenter RCTs, longitudinal cohorts, and causal inference frameworks such as instrumental variable analysis or Mendelian randomization. Incorporating directed acyclic graphs (DAGs) can help identify and control for key confounders, including diet, antibiotics, PPIs, metformin, and baseline medications (Forslund et al., 2015; Freedberg et al., 2015; Weersma et al., 2020).

HF subtypes should also be pre-stratified (e.g., HFrEF vs. HFpEF, acute vs. chronic, metabolic or obesity-related phenotypes) to improve external validity. Routes of drug administration-oral, enteric-coated, sustained-release, or intravenous-must be recognized as essential covariates because they determine the degree of drug-microbiota interaction and metabolic exposure (Zhang X et al., 2021).

### 5.2 Technical standardization and data integration in microbiome research

Technical variability remains a major obstacle to reproducibility in microbiome studies. Differences in sampling methods, extraction protocols, sequencing platforms, and bioinformatic pipelines complicate cross-study comparison. Adoption of standardized frameworks such as the STORMS reporting checklist (Mirzayi et al., 2021), MIxS standards (Jorge et al., 2022), and FAIR data principles is critical. Key methodological parameters, including sample matrix (fecal vs. mucosal vs. oral), preservation conditions, batch-effect control, and contamination management, must be explicitly reported.

Reliance on relative abundance alone can introduce compositional biases, so integrating absolute quantification approaches (qPCR, FISH, flow cytometry combined with 16S/metagenomics) is essential for accurately defining microbial load and drug-relevant dose-response relationships (Gronich et al., 2025; Props et al., 2017; Tkacz et al., 2018; Vandeputte et al., 2017; Zong et al., 2022). Future progress requires harmonized analytical pipelines and integrated analysis of metagenomic, metabolomic, and pharmacokinetic datasets. Machine learning applications should prioritize interpretability and reproducibility, with fully transparent training, validation, and calibration protocols and open-source code supported by independent external validation sets. These approaches can ultimately generate reliable predictive models for microbiota–drug interactions and clinical outcomes.

### 5.3 Regulatory and ethical considerations in microbiome-based therapies

As pharmacomicrobiomics moves toward clinical implementation, regulatory and ethical challenges become increasingly important. Novel interventions, such as FMT, microbial metabolite inhibitors, and extensive genetic or metagenomic profiling, require careful evaluation of safety, efficacy, and long-term risks.

Future drug development should incorporate microbiome assessments during both preclinical and clinical stages. Drug labels or pharmacokinetic documentation may eventually include microbiota-related interaction profiles, enabling more rational co-therapy and dose adjustment.

Ethical considerations include informed consent, data privacy, and transparent handling of microbiome and genomic information. Safeguards are needed to prevent discrimination or stigmatization based on microbiome signatures. Ensuring health equity is essential so that personalized microbiome-based therapies do not become accessible only to select populations. Regulatory frameworks must therefore support innovation while promoting equitable and responsible adoption.

### 5.4 Multidisciplinary collaboration and future research pathways

Advancing pharmacomicrobiomics toward precision HF care will require systematic progress across several domains. Because microbial composition varies widely by diet and population background (Wilson et al., 2020), comprehensive multi-omics integration, including metagenomics, metabolomics, pharmacogenomics, and clinical phenotyping, is crucial for identifying microbe-host pathways that influence HF progression, therapeutic efficacy, and adverse reactions.

Building upon mechanistic insights, AI and machine learning tools can generate composite models that integrate microbiome features, host genetics, and clinical indicators. Such models could enable early prediction of treatment response, toxicity risk, and prognosis, and provide data-driven support for personalized HF therapy.

Innovation in microbiota-targeted interventions should also be pursued. Promising directions include precision dietary optimization (e.g., fiber or polyphenol tailoring), next-generation probiotics, defined clinical pathways for FMT, and development of microbial metabolic modulators such as TMAO inhibitors or SCFA receptor agonists. These approaches require validation through large, well-designed RCTs assessing both clinical endpoints and long-term safety. Complementary real-world data and registry studies can help evaluate generalizability, adherence, and cost-effectiveness.

Ultimately, deep interdisciplinary collaboration, linking cardiology, microbiology, pharmacology, nutrition, computational science, and bioengineering, is essential. Building standardized functional analysis platforms and shared clinical databases will be key to supporting mechanistic discovery and clinical translation. A streamlined bench-to-bedside pathway will accelerate the adoption of microbiome-guided precision therapy for HF and move the field from conceptual potential to clinical reality.

This diagram outlines the key challenges and future directions in microbiome research related to HF. The first challenge emphasizes the need for more high-quality randomized studies to address research gaps and strengthen the evidence base. The second challenge focuses on the technical standardization and integration of microbiome data, highlighting the need for standardized procedures to ensure consistent and reproducible results. The third challenge involves the assessment of microbiome research, with an emphasis on regulatory and ethical considerations related to gut microbiome interventions. Finally, the fourth challenge underscores the importance of multidisciplinary cooperation, with a call for greater collaboration across fields to accelerate microbiome-based therapies in HF management.

## 6 Conclusion

HF remains a major global public health challenge. Pharmacological therapies have improved outcomes, but large differences in treatment response and drug-related adverse effects still persist among patients. Pharmacomicrobiomics offers a new way to understand these differences, because growing evidence shows that the gut microbiota and its metabolites participate in HF pathogenesis and progression and can also influence drug metabolism, bioavailability, and pharmacodynamic activity. These effects may shape both therapeutic efficacy and safety.

This review proposes an integrative pharmacomicrobiomics framework for heart failure, linking microbial dysbiosis, drug-microbiota interactions, and microbiome-guided therapeutic strategies. It summarizes the bidirectional interactions between the gut microbiome and major HF medications, including SGLT2 inhibitors, digoxin, ACEIs, ARBs, ARNIs, β-blockers, diuretics, and MRAs. It highlights how microbiome-based biomarkers may help predict therapeutic response, explain treatment failure and adverse drug reactions, and guide individualized dose adjustment and clinical decision-making. It also discusses current advances and challenges in microbiome-targeted interventions, such as dietary strategies, microbial therapeutics, and the development of new drugs that target microbial pathways.

Despite these promising insights, important limitations remain. Most existing studies are observational, so causality cannot be confirmed. Sample sizes are often small, follow-up periods are short, and methodological standards vary widely. These gaps restrict clinical translation. In the future, integrating multi-omics technologies with artificial intelligence (AI)-based prediction models may help accelerate the transition of pharmacomicrobiomics from concept to clinical application.

Through broad collaboration across cardiology, microbiology, pharmacology, nutrition, and data science, standardized research frameworks and prospective intervention trials can support the development of microbiome-informed precision therapy for HF, and may offer new ways to improve long-term outcomes and quality of life.

## Glossary

ACEIs: Angiotensin-converting enzyme inhibitors
ADME: Absorption, Distribution, Metabolism, and Excretion
ADRs: Adverse drug reactions
AI: Artificial intelligence
ARBs: Angiotensin receptor blockers
ARNIs: Angiotensin receptor neprilysin inhibitors
BNP: B-type Natriuretic Peptide
BP: Blood pressure
Cgr: Cardiac glycoside reductase
DAGs: Directed acyclic graphs
DMB: 3,3-Dimethyl-1-butanol
DM: Diabetic Mellitus
FAIR: Findable, Accessible, Interoperable, and Reusable
FISH: Fluorescence *in situ* hybridization
FMO3: Flavin-containing monooxygenase 3
FMT: Fecal microbiota transplantation
FXR: Farnesoid X receptor
GPCRs: G protein-coupled receptors
HF: Heart failure
HFpEF: Heart failure with preserved ejection fraction
HFrEF: Heart failure with reduced ejection fraction
HIF-2α: Hypoxia-inducible factor 2α
IL-23R: Interleukin-23 receptor
I/R: Ischemia/Reperfusion
IRI: Ischemia-Reperfusion Injury
MI: Myocardial Infarction
MIxS: Minimum Information about any (x) Sequence
ML: Machine learning
MR: Mendelian randomization
MRAs: Mineralocorticoid receptor antagonists
NAPE: N-acyl-phosphatidylethanolamine
NHFA: National Heart Failure Audit
NO: Nitric oxide
Olfr78: Olfactory receptor 78
PAGln: Phenylacetylglutamine
PBPK: Physiologically based pharmacokinetic
PK: Pharmacokinetic
PPIs: Proton pump inhibitors
qPCR: Quantitative polymerase chain reaction
RAAS: Renin-angiotensin-aldosterone system
RCTs: Randomized controlled trials
RWD: Real-world data
SCFAs: Short-chain fatty acids
sGC: Soluble guanylate cyclase
SGK1: Serum/Glucocorticoid regulated kinase 1
SGLT2: Sodium-glucose cotransporter 2
TGR5: Takeda G protein-coupled receptor 5
Th17: T helper 17 cells
TMA: Trimethylamine
TMAO: Trimethylamine N-oxide
Treg: Regulatory T cells
